# Coherent amplification of radiation from two phase-locked Josephson junction arrays

**DOI:** 10.3762/bjnano.13.119

**Published:** 2022-12-06

**Authors:** Mikhail A Galin, Vladimir M Krasnov, Ilya A Shereshevsky, Nadezhda K Vdovicheva, Vladislav V Kurin

**Affiliations:** 1 Institute for Physics of Microstructures RAS, 603950 Nizhny Novgorod, Russia; 2 Department of Physics, Stockholm University, AlbaNova University Center, SE-10691 Stockholm, Sweden

**Keywords:** coherent radiation, Josephson junction arrays, numerical modelling, single-strip line, synchronization

## Abstract

We analyze experimentally and theoretically mutual phase locking and electromagnetic interaction between two linear arrays with a large number of Josephson junctions. Arrays with different separation, either on the same chip or on two separate substrates are studied. We observe a large coherent gain, up to a factor of three, of emitted power from two simultaneously biased arrays, compared to the sum of powers from two individually biased arrays. The phenomenon is attributed to the phase locking of junctions in different arrays via a common electromagnetic field. Remarkably, the gain can exceed the factor of two expected for a simple constructive interference of two oscillators. The larger gain is explained by an additional consequence of mutual interaction between two large arrays. Mutual phase locking of large arrays does not only result in constructive interference outside the arrays, but also improved synchronization of junctions inside each array. Our conclusion is supported by numerical modelling.

## Introduction

A Josephson junction (JJ) has the unique ability to transform an applied constant voltage *V* into electromagnetic (EM) oscillations. The fundamental Josephson frequency, *f*_J_, is connected to *V* via the ac-Josephson relation, *hf*_J_ = 2*eV*, where *h* is the Planck constant and *e* is the elementary charge. Josephson generation occurs up to the superconducting gap voltage. Therefore, *f*_J_ can be up to about 1 THz for low-*T*_c_ JJs [1] and can reach tens of terahertz for high-*T*_c_ JJs [2–3]. Thus, a JJ has the potential to be the basis of compact, continuous-wave and tunable terahertz generators, which would facilitate solving the problem of so-called “THz gap” [4].

A single JJ emits only a very small off-chip power, typically in the picowatt range. To enhance it to a practical level of about 1 mW, it is necessary to combine many equivalent junctions in an array [5]. However, synchronization of a large number of radiation sources is a serious electrodynamical problem. This problem can be resolved for a group of JJs that extends in subwavelength dimensions. Such a configuration is realized for intrinsic JJs formed in a BiSCCO crystal where almost 700 JJs are localized within 1 μm [6]. The generation in a wide frequency range of 1–11 THz has been demonstrated from BiSCCO mesas containing up to 250 JJs [2]. A maximal emission of about 1 μW corresponds to in-phase cavity modes in the mesas, indicating the coherent superradiant nature of the emission.

The arrays based on intrinsic JJs suffer from overheating, which impedes a raise of radiation power. This problem manifests considerably less in discrete JJ arrays. Modern lithographic technologies allow for fabricating discrete JJs with dimensions down to the sub-100 nm scale [7]. However, the size of very large arrays with thousands of JJs may exceed the radiation wavelength. For such superwavelength systems, delay effects become dominant for synchronization. To reach the radiation power maximum, JJs should be synchronized with the EM mode excited within the resonator. Such large JJ arrays become similar to a laser where the junctions play the role of atoms in an active medium. The advantage of large JJ arrays working similar to lasers is discussed in more detail in [8]. The resonator can be a cavity of the JJs itself [2], an electrode with embedded JJs [9], or the dielectric substrate on which the JJ array is arranged [10].

Coherent superradiant amplification of emitted power is caused by a constructive interference of EM fields from phase-locked oscillators [11]. For two oscillators, the EM field in the far-field maximally doubles. Hence, the superradiant power, proportional to the square of the EM field, is at most four times larger than that from a single oscillator. For incoherent emission from two unlocked oscillators the power just adds up and is twice the power from a single oscillator. Therefore, the total superradiant power gain for two oscillators, defined as the ratio of coherent-to-incoherent emission, is at most two. For *N* oscillators, the supperradiant power increase is at most *N*^2^ times the power from one oscillator and the superradiant gain factor is at most *N*. For large *N*, this could greatly enhance the emitted power. This is the main motivation for the development of Josephson oscillators based on arrays with many JJs [2–3,6,9–10,12–15].

Resonant modes formed along five straight electrodes with niobium JJs have been directly visualized recently using low-temperature scanning laser microscopy [13]. The scans revealed that the standing waves can provide the global coupling of all junctions in the array, that is, extended parts of the array can interact with each other. This generates the two-dimensional resonant mode that should lead to the increased output power. Therefore, along with the interaction between individual JJs, there is also a mutual coupling between different arrays (including JJs and electrodes) [14]. In the ideal case of two perfectly phase-locked arrays with *N* JJs each, the total superradiant power is proportional to 4*N*^2^, which is two times larger than the sum of powers from two incoherent arrays. Therefore, such arrays can be considered as individual oscillators, and the gain factor for two phase-locked arrays equals two. Yet, the physics of inter-array coupling is much more complicated. In reality, synchronization between junctions in each array is not perfect due to the insufficient amplitude of the resonant mode. In this case, resonant coupling of two arrays may improve the state within each array. As we will show, this could increase the gain factor well above a factor of two. The goal of our work is to study inter-array coupling and its manifestations.

In this work we study the interaction between two linear arrays of Nb/NbSi/Nb JJs. The arrays have a single-line geometry with 332 or 380 JJs embedded in a straight electrode. We analyze the mutual interaction between two independently biased arrays oriented parallel to each other. First, we study arrays on the same chip for different distances of 4 and 238 μm between them. Then, we consider two arrays on different chips, stacked on top of each other. We perform simultaneously the measurement of current–voltage characteristic (IVC) and bolometric analysis of the emitted radiation. In all cases, we observe clear signatures of inter-array interaction. They occur when both arrays are biased at the same voltage and oscillate at the same frequency, coinciding with one of the cavity modes in the array electrodes. This leads to a profound enhancement of resonant step amplitudes in the IVCs of the arrays, indicating that the state of one array is strongly affected by oscillations in another array. The inter-array coupling is manifested by a significant amplification of emitted power with a gain factor of up to three. It is well above the factor of two expected in the simple case of bare coherent superposition of oscillations. This result points out that phase locking of oscillations in the two arrays not only leads to coherent amplification of radiation. It also can improve the synchronization inside each array. The latter effect removes the limit of two for the gain factor. Finally, for better understanding, we performed numerical simulations of the inner dynamics for two interacting arrays. Our simulations confirm that two arrays can be phase-locked by a common EM field. They also provide estimation of the resulting superradiant gain. The performed experimental investigations and numerical calculations can give new ideas about the design of discrete JJ arrays that would provide more effective synchronization of JJs in order to get an output power sufficient for practical applications.

## Experimental

### Samples

We study samples containing one or several straight strips with embedded Nb/NbSi/Nb overlap JJs connected in series. The samples were fabricated by Oliver Kieler (Braunschweig, Germany) and were measured in AlbaNova University Center (Stockholm, Sweden). The fabrication is a self-aligning process using e-beam lithography and reactive ion etching [16–17]. Similar arrays were studied earlier in [9,12–13], where additional information about sample characterization can be found.

**Figure 1:**
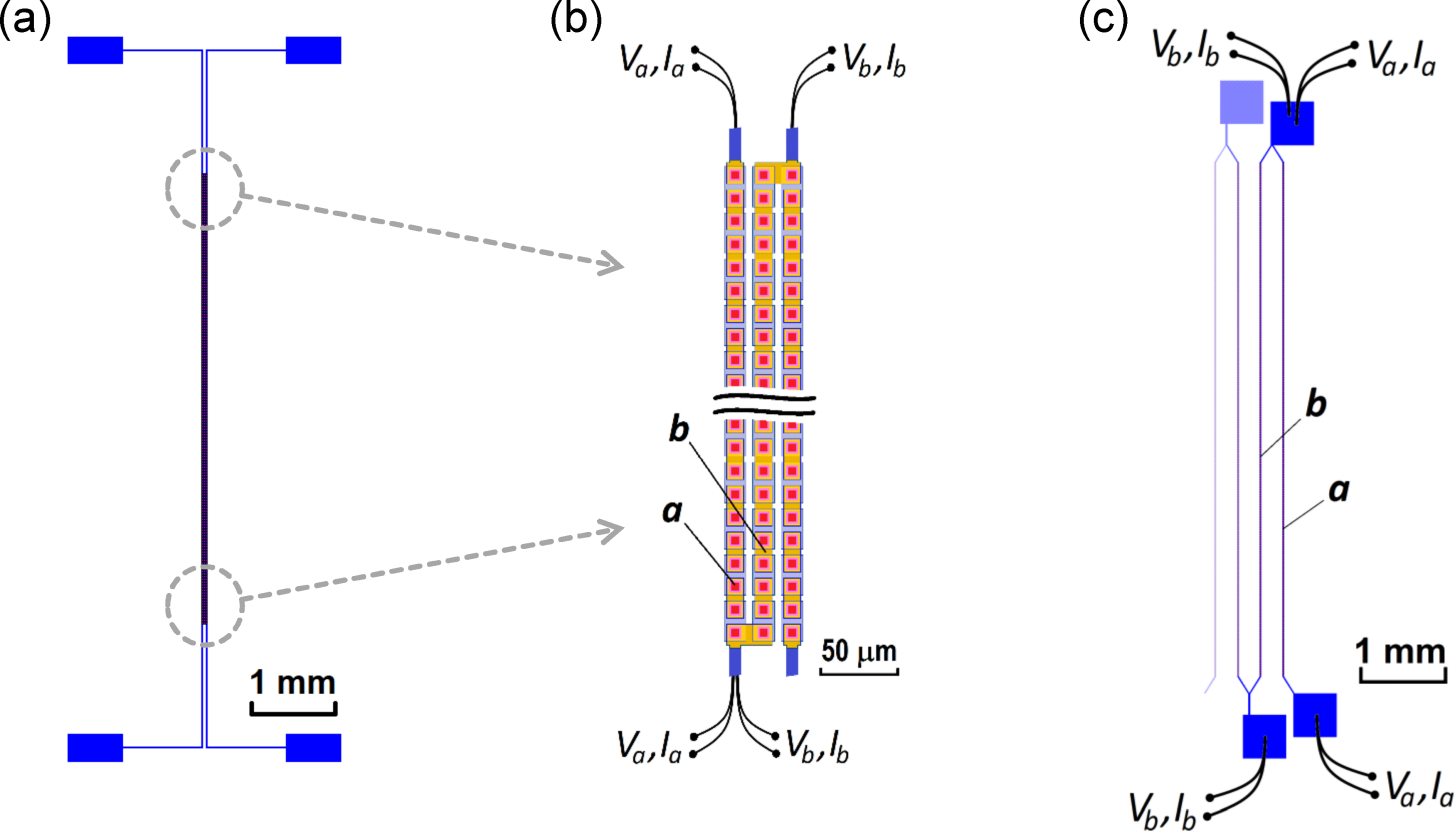
(a) Geometry of sample-1 with 332 JJs in each of the three linear arrays. (b) Two enlarged fragments of sample-1. Red squares represent the JJs, which are between the top (orange rectangles) and the bottom (partly covered blue rectangles) niobium electrodes. (c) Geometry of the right part of sample-2 with 380 JJs in each linear array. In the measurements with either of the samples, the outer array-a was biased by a sweep current (*V*_a_, *I*_a_) while the adjacent inner array-b was biased at fixed voltage *V*_b_ and current *I*_b_ by another current source. The points of source connections are depicted schematically.

Figure 1a,b shows the layout of “sample-1”. It has been fabricated on a 1 × 1 cm^2^ silicon substrate with the thickness 0.38 mm. It contains three closely located straight strips with a separation of only 4 μm. Each strip has the length *L* = 5 mm and the width *w* = 14 μm and contains 332 JJs distributed uniformly along the strip. The junction area is 8 × 8 μm^2^. Contact electrodes are connected to each strip, allowing for independent biasing of each of these three arrays. Below, we will analyze the interaction between the leftmost “array-a” biased with a variable dc current and “array-b” in the middle biased with a fixed current (Figure 1b).

Figure 1c shows the layout of “sample-2”. It has a significantly larger separation of 238 μm between the adjacent linear arrays. In total, it contains 17 similar lines with 380 JJs and a total length of *L* = 5.7 mm. The junction area is 6 × 6 μm^2^. Below, we will show data for the case when the rightmost “array-a” is biased with a variable dc current and the nearby “array-b” is biased with a fixed current.

Sample-1 and sample-2 were used for on-chip analysis where two linear arrays are placed on the same substrate. We also present data for off-chip synchronization. To this end, two linear arrays were stacked on top of each other.

### Radiation detection

An InSb bolometer is used for the detection of Josephson radiation. The detector and measurement procedure are the same as described in [9,12], where additional information can be found. The bolometer is based on a high-purity n-doped InSb crystal with dimension of 2–3 mm, which is placed approximately 0.5 cm above the array. The absorbed radiation causes an increase of charge carriers in the conduction band and leads to a decrease of the dc voltage at a fixed bias current. Therefore, we take the negative change of the dc voltage of the bolometer Δ*U* as a measure of the absorbed power, that is, Δ*U* = *U*_0_ − *U >* 0 where *U*_0_ and *U* are the voltages on the crystal in absence and in presence of the radiation, respectively. All measurements were performed in a liquid helium dewar at a temperatures *T* 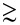 4.2 K both for the samples and the detector. The calibrated responsivity of the detector at this temperature was estimated as ≃300 V/W.

## Results

**Figure 2:**
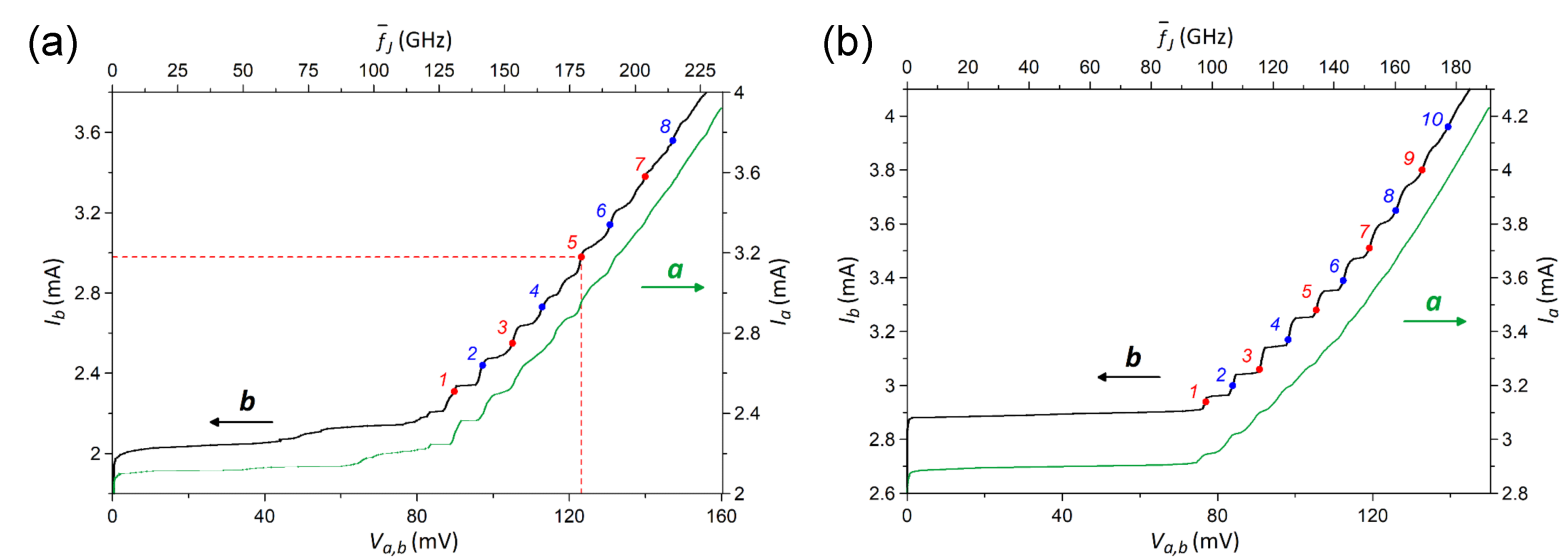
IVCs of outer array-a (rigth axis) and adjacent inner array-b (left axis) of sample-1 (a) and sample-2 (b). The enumerated points correspond to the biasing of the inner array with currents of *I*_b_ = 2.31 (1), 2.44 (2), 2.55 (3), 2.73 (4), 2.98 (5), 3.14 (6), 3.38 (7), and 3.56 mA (8) for sample-1 (a) and with currents of *I*_b_ = 2.94 (1), 3 (2), 3.06 (3), 3.17 (4), 3.28 (5), 3.39 (6), 3.51 (7), 3.65 (8), 3.8 (9), and 3.96 mA (10) for sample-2 (b). The case of biasing at the indicated point 5 in panel (a) is discussed in Figure 3a,b.

Figure 2a shows the individually measured IVCs of array-a and array-b of sample-1 (Figure 1a,b). The critical current in both arrays is *I*_c_ = 2.0–2.1 mA and the characteristic frequency, estimated within the resistively shunted junction (RSJ) model, is in the range of *f*_c_ ∼ 100–120 GHz. Figure 2b shows similar data for sample-2. Here, for both arrays, *I*_c_ ≈ 2.9 mA and *f*_c_ ∼ 80–100 GHz. Although the area of junctions in sample-2 is smaller, the critical current is slightly larger than in sample-1 due to higher doping of the NbSi interlayers. Note that the abrupt transition from a superconductive to a resistive state observed in all IVCs is typical for niobium junctions with medium doping Si interlayer 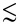
11% [18].

Resonant steps at similar voltages are observed for both pairs of arrays. As shown earlier [9,13], they are caused by standing wave (cavity mode) resonances in the whole length of strips of JJs. However, for both samples the steps are more pronounced in the inner array-b than in the outer array-a. Presumably, this is due to a more beneficial EM environment for the inner strip, which has two adjacent strips on both sides operating as additional single-strip line resonators (see Figure 1).

In Figure 2a, we marked bias points 1–8 in array-b, at which detailed measurements are reported below for sample-1. Using the value of EM wave speed along a single-strip line obtained in [13], we can estimate the corresponding numbers of cavity modes *m* = 11–18. Similarly, bias points 1–10 in Figure 2b are used for analysis of sample-2. According to our estimation, they correspond to numbers of cavity modes *m* = 10–19.

### Synchronization of two arrays on the same chip

In this work, we aim to study the EM interaction between two independently biased arrays. For the on-chip measurement, the bias current in the outer array-a *I*_a_ was varied while the middle/inner array-b was biased at fixed values of current *I*_b_ = const. All other arrays remain unbiased and, therefore, inactive. Thus, we measure the dc voltage of array-b *V*_b_ and the full IVC of array-a *V*_a_(*I*_a_). Simultaneously, the radiation signal Δ*U*_ab_(*V*_a_, *V*_b_) is detected. The dc bias point of array-b (*I*_b_, *V*_b_) is chosen at one of the resonant steps. These points are marked by numbers in the IVCs of Figure 2.

#### Synchronization of closely spaced arrays (sample-1)

**Figure 3:**
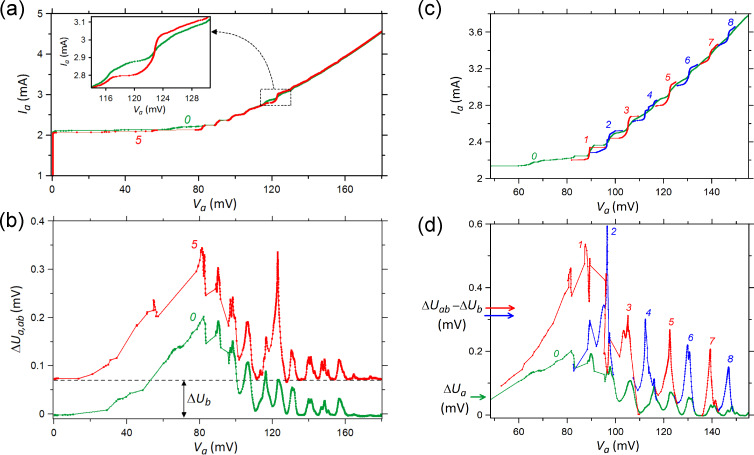
(a, b) IVCs of the outer array-a in sample-1 (a) and corresponding bolometer signal (b) when the inner array-b is unbiased (curves 0) and biased at *I*_b_ = 2.98 mA, *V*_b_ = 123 mV, which corresponds to point 5 indicated in Figure 2a (curves 5). The inset in panel (a) shows an enlarged fragment of the IVCs at *V*_a_ ∼ *V*_b_ with the current step, which becomes greater and more distinct due to the EM interaction between the two arrays. The value Δ*U*_b_ in panel (b) is the radiation signal derived from array-b at unbiased array-a. (c, d) The data set for the IVCs of the outer array-a (c) and for the bolometer signal (d). Curves 0 are derived at an unbiased inner array-b while curves 1–8 correspond to the biasing of array-b at the points indicated in Figure 2a. The curves 1–8 are the fragments of IVCs and bolometer signals in the ranges where significant changes relative to the curves 0 are observed.

We start with sample-1, which has the shorter separation between the strips. Figure 3a shows two IVCs of array-a. The green IVC is measured with a passive array-b, *V*_b_ = 0, and the red is measured with a fixed bias *I*_b_ = 2.98 mA and *V*_b_ = 123 mV, corresponding to the bias point 5 in Figure 2a. The inset shows a close-up of the voltage range *V*_a_ ∼ *V*_b_, which demonstrates that oscillations in array-b lead to a pronounced enhancement of the resonant step in array-a at *V*_a_ ≈ *V*_b_ while other steps are practically unaffected. The differential resistance in the center of this step *R*_d_ decreases by a factor of four, from *R*_d_ = 16 Ω to *R*_d_ = 4 Ω. Since the step amplitude reflects (approximately proportionally) the amplitude of the EM field in the cavity mode, this clearly demonstrates that the active array-b amplifies the EM oscillation amplitude in array-a under the condition *V*_a_ ≃ *V*_b_. This means almost exact equality of the mean Josephson frequencies averaged over all JJs 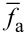 ≃ 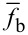 ≈ 179 GHz, which is the necessary condition for phase locking.

In Figure 3b, we present results of the radiation detection measured simultaneously with the IVCs in Figure 3a. The lower green curve shows the detector signal Δ*U*_a_(*V*_a_, *V*_b_ = 0) as a function of the voltage *V*_a_ in array-a, for the unbiased array-b, *V*_b_ = 0. It represents the emission power solely from array-a. The upper red curve shows similar data, Δ*U*_ab_(*V*_a_, *V*_b_), when array-b is biased to the point 5 in Figure 2a. It is seen that here Δ*U*_ab_(*V*_a_ = 0) = 0.07 mV. This signal offset represents the emission Δ*U*_b_(*V*_b_) from array-b alone. It can be seen that the shapes of the two curves in Figure 3b are quite similar. At almost all *V*_a_, they simply differ by a constant offset, Δ*U*_ab_(*V*_a_, *V*_b_ = const) ≃ Δ*U*_a_(*V*_a_, *V*_b_ = 0) + Δ*U*_b_(*V*_a_ = 0, *V*_b_ = const), as indicated by the dashed horizontal line. This implies that, usually, the powers from the two arrays simply add up, which is typical for the incoherent state. However, a remarkable peak is observed when the voltages of the two arrays practically coincide, *V*_a_ ≃ *V*_b_ = 123 mV. At this point, Δ*U*_ab_(*V*_a_ ≃ *V*_b_) = 0.34 mV, which is 2.4 times larger than the sum of individual arrays Δ*U*_a_(*V*_b_) + Δ*U*_b_(*V*_b_) ≃ 2Δ*U*_b_(*V*_b_) = 0.14 mV. To quantify this effect, we consider the gain factor


[1]

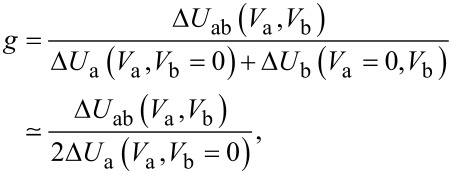



which describes the coherent superradiant amplification of the radiation power.

In Figure 3c,d, we show a similar analysis for all explored bias points in array-b. Figure 3c shows the IVC of array-a *I*_a_(*V*_a_) without bias in array-b (green line) and portions of the IVCs at bias points 1–8 of array-b indicated in Figure 2a (red and blue curves). Here, we show eight fragments of separately measured IVCs close to the condition of phase locking *V*_a_ ∼ *V*_b_. It can be seen that at all bias points, *V*_b_, a strong enhancement of the resonant step in array-a occurs compared to the case without bias, *V*_b_ = 0. This is particularly clear for higher bias points 7 and 8, for which the steps without bias in array-b are barely visible, but with bias they are well developed.

Figure 3d shows the detector response Δ*U*_a_(*V*_a_, *V*_b_) measured simultaneously with the IVCs from Figure 3c. The lower green curve is measured at an unbiased array-b. The upper red/blue curves correspond to bias points 1–8 in array-b (Figure 2a). At low bias, bias points 1–3, we observe a multimode excitation, that is, some gain occurs even at modes adjacent to *V*_b_. At higher bias, single mode amplification takes place, as for the bias point 5 discussed above (Figure 3b). The gain factors for all eight bias points are given in Table 1. The highest gain is observed at point 7 with *g* = 2.9.

**Table 1:** Gain factor from the interaction of outer and inner arrays in sample-1.

No. of step/bias point	1	2	3	4	5	6	7	8

*I*_b_, mA	2.31	2.44	2.55	2.73	2.98	3.14	3.38	3.56
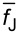 , GHz	127.5	140.7	153.2	163.7	178.4	189.2	202.9	213.8
*g*	1.8	1.9	1.9	1.6	2.3	2.1	2.9	2.5

#### Synchronization of more distant arrays (sample-2)

**Figure 4:**
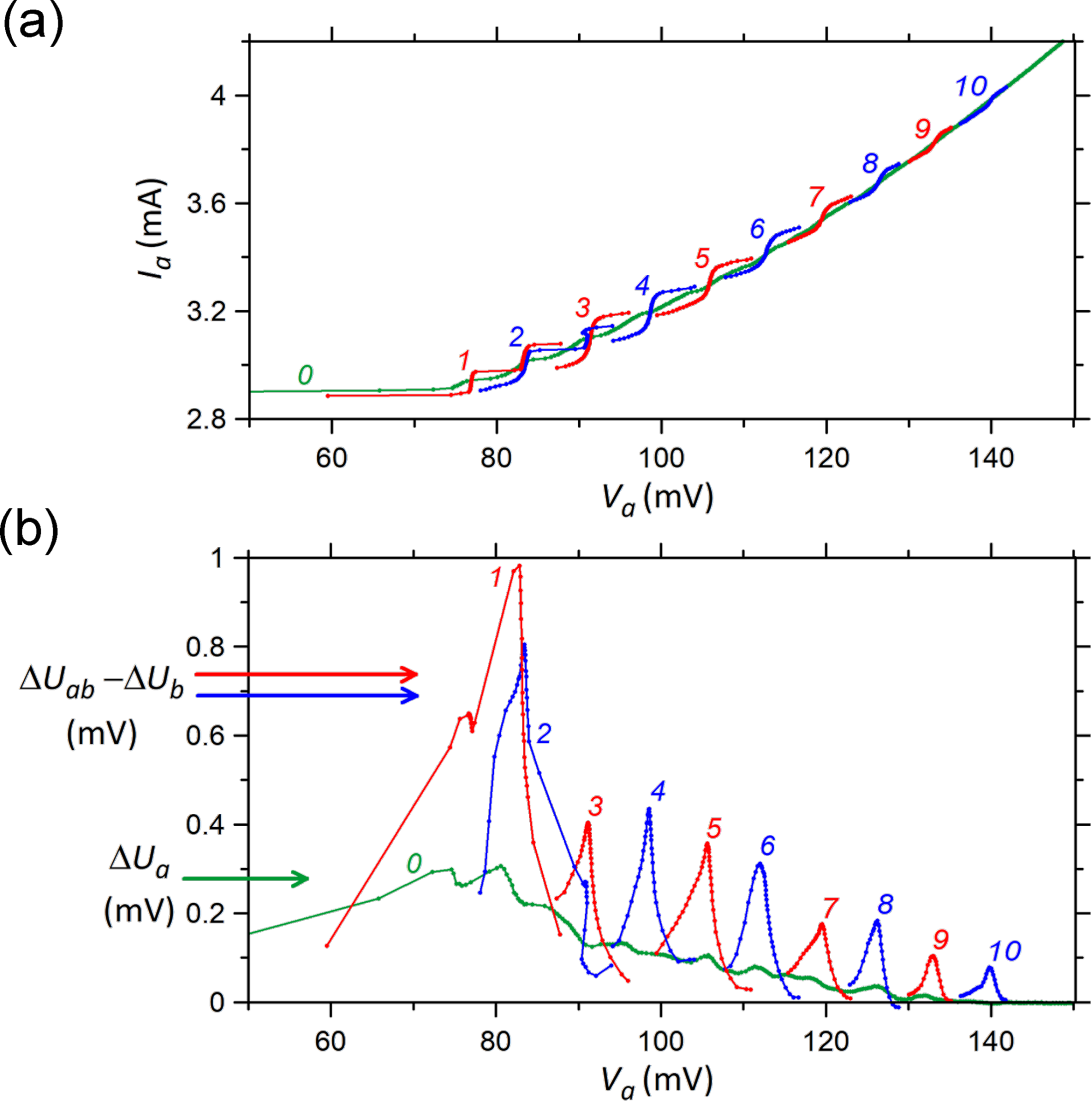
The data set for the IVCs of the outer array-a in sample-2 (a) and for the corresponding bolometer signal (b). Curves 0 are derived at unbiased inner array-b while curves 1–10 correspond to biasing of this array to the points indicated in Figure 2b. The curves 1–10 are the fragments of IVCs and bolometer signals in the ranges where significant changes relative to the curves 0 are observed. The value Δ*U*_b_ in panel (b) is the radiation signal derived from array-b at unbiased array-a.

Next, we consider sample-2 with significantly larger separation between the arrays, namely 238 μm. Figure 4a shows IVCs of the outer array-a. The green curve shows the result without bias in the inner array-b. The blue and red curves show results with bias at points 1–10 indicated in Figure 2b. Comparable to sample-1, Figure 3c, we also can see a significant enhancement of steps in the array at *V*_a_ ≈ *V*_b_. Figure 4b shows the detector response measured simultaneously with the IVCs from Figure 4a. A significant enhancement of emission occurs practically under the condition of phase locking, *V*_a_ ≃ *V*_b_. The corresponding gain factors are listed in Table 2. They are only slightly smaller than those for sample-1, indicating that the inter-array coupling is not a short-range phenomenon.

**Table 2:** Gain factor from the interaction of outer and inner arrays in sample-2.

No. of step	1	2	3	4	5	6	7	8	9	10

*I*_b_, mA	2.94	3	3.06	3.17	3.28	3.39	3.51	3.65	3.8	3.96
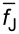 , GHz	97.9	106.8	115.6	124.9	134.1	143.0	151.5	160.2	168.9	177.4
*g*	1.5	1.8	1.8	1.9	1.8	2.0	1.6	1.9	2.0	2.6

### Off-chip synchronization

**Figure 5:**
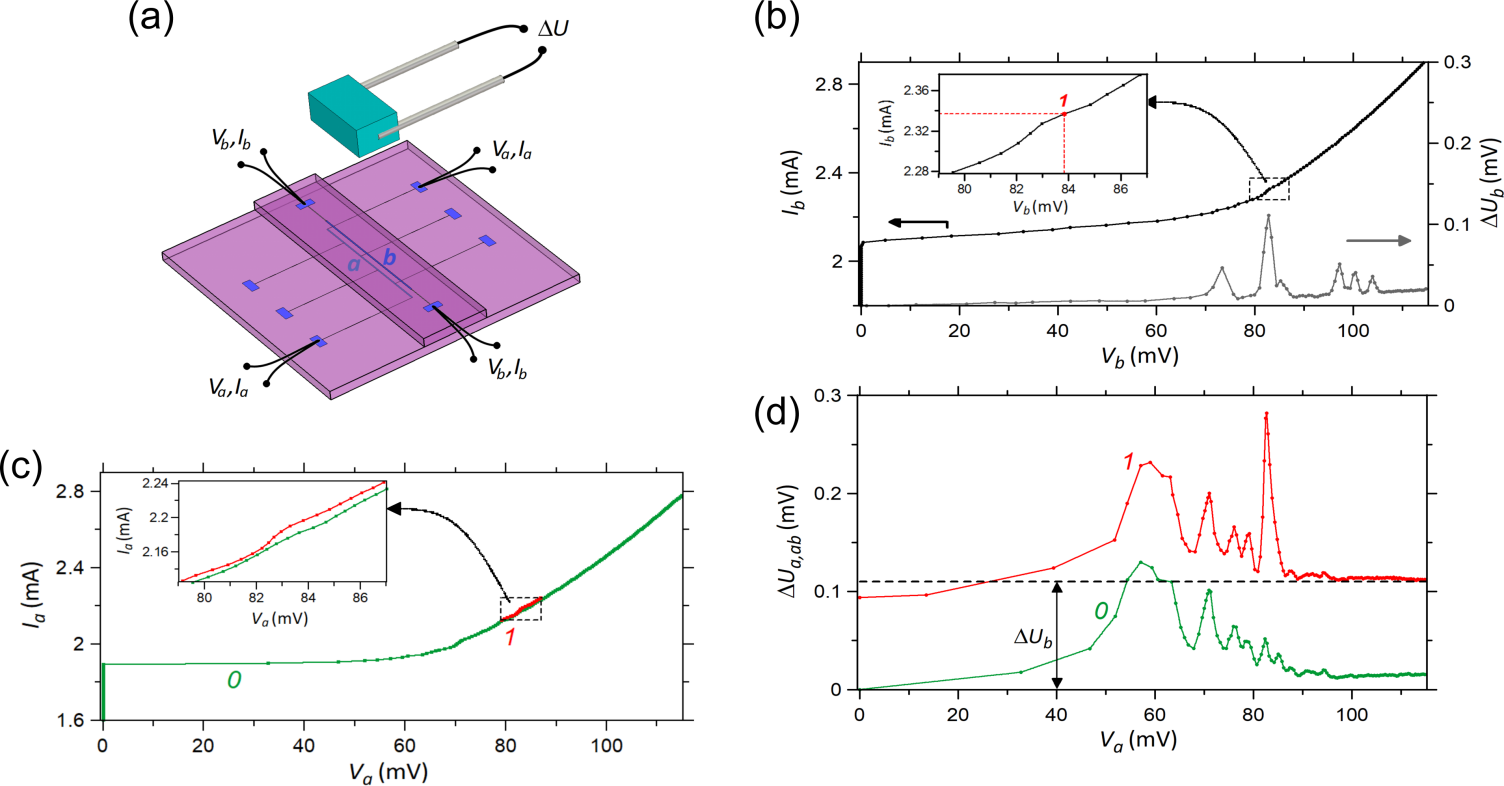
(a) View of the measurement scheme with two JJ arrays on different substrates formed in a stack. Each array is a straight strip with 332 JJs. In the measurements, bottom array-a was biased by a sweep current (*V*_a_, *I*_a_) while top array-b was constantly biased (*V*_b_, *I*_b_) by another current source. Above the stack is the InSb bolometer, in which the signal Δ*U* caused by the Josephson radiation from the arrays is measured. (b) IVC of array-b (left axis) and corresponding bolometer signal (right axis) when array-a is unbiased. The inset shows the enlarged fragment of the IVC with some weak current step where the indicated bias point *I*_b_ = 2.34 mA, *V*_b_ = 84 mV was chosen for the measurement with two biased arrays. (c, d) IVC of the bottom strip (c) and bolometer signal (d) when array-b is unbiased (curves 0) or biased at the point *V*_b_, *I*_b_ (curves 1). The inset in panel (c) shows the enlarged fragment of IVCs at *V*_a_ ∼ *V*_b_ with the weak current step that appears due to the EM interaction between two strips. The value Δ*U*_b_ in panel (d) is the radiation signal derived from array-b at unbiased array-a.

The third series of measurements was performed using arrays at two different substrates. The substrates were stacked on top of each other, as sketched in Figure 5a. To facilitate access to the contact pads of the bottom array, the substrate of the top array was trimmed to a width of about 3 mm. The arrays are similar to those in sample-1, but with a different shape of connecting electrodes (cf. Figure 1a,b and Figure 5a), which does not influence the measurements. The distance between two arrays is approximately equal to the thickness of substrate plus the glue layer and, possibly, a slight misalignment in the lateral direction. Overall, it is about 0.4 mm through the silicon substrate. During measurements, the bottom array-a is biased with a variable dc current and the top array-b is biased with a fixed current. To obtain the most prominent effect, we slightly increased the temperature of the stack to *T* = 4.4 K by placing it above the surface of liquid helium.

Figure 5b shows the IVC of the individually biased top array-b together with the simultaneously measured detector signal. The IVC has the characteristic parameters *I*_c_ = 2.1 mA and *f*_c_ ≈ 120 GHz. The maximum radiation signal Δ*U*_b_ = 0.11 mV is observed at *I*_b_ = 2.34 mA, *V*_b_ = 84 mV, which corresponds to the mean Josephson frequency 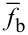 = 121 GHz. At this bias point, a weak current step is observed (Figure 5b, inset), which actually is the only one in the whole IVC curve. This bias point is chosen for the subsequent measurements.

In Figure 5c, the green curve represents the individual IVC of array-a without bias in array-b. The red curve shows the part of IVC with bias in array-b at the maximum emission point 1 indicated in the inset in Figure 5b. This IVC has *I*_c_ = 1.9 mA and *f*_c_ ≈ 100 GHz, which are close to the values for array-b. Figure 5d shows the simultaneously measured detector signal. The lower green curve represents the emission signal from the individual array-a, Δ*U*_a_(*V*_a_, *V*_b_ = 0). Note that it has a clearly different shape compared to Δ*U*_b_(*V*_a_ = 0, *V*_b_) shown in Figure 5b. The upper red curve represents the combined emission, Δ*U*_ab_(*V*_a_, *V*_b_). The general behavior is quite similar to that found in the on-chip experiments. A sharp peak is revealed under the condition of phase locking, *V*_a_ = *V*_b_, with Δ*U*_ab_ = 0.28 mV. It is considerably larger than the sum Δ*U*_a_ + Δ*U*_b_ = 0.17 mV. The gain factor is *g* = 1.7. Note that, in contrast to on-chip measurements, Δ*U*_a_ ≠ Δ*U*_b_, which can depend on the difference in position of the two arrays with respect to the detector, different geometries of substrates and connecting electrodes, and the stacking arrangement of the samples.

The obtained results show that the coupling between JJ arrays can be realized due to EM waves propagating inside the substrate. Note that the distance between the arrays is close to the half wavelength in the substrate, λ_Si_/2 = 
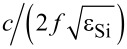
 (ε_Si_ = 11.9 is the dielectric permittivity of silicon). Under this condition, the fundamental resonant mode can be excited in the substrate between the arrays. This condition is beneficial for inter-array coupling.

## Numerical Calculations

**Figure 6:**
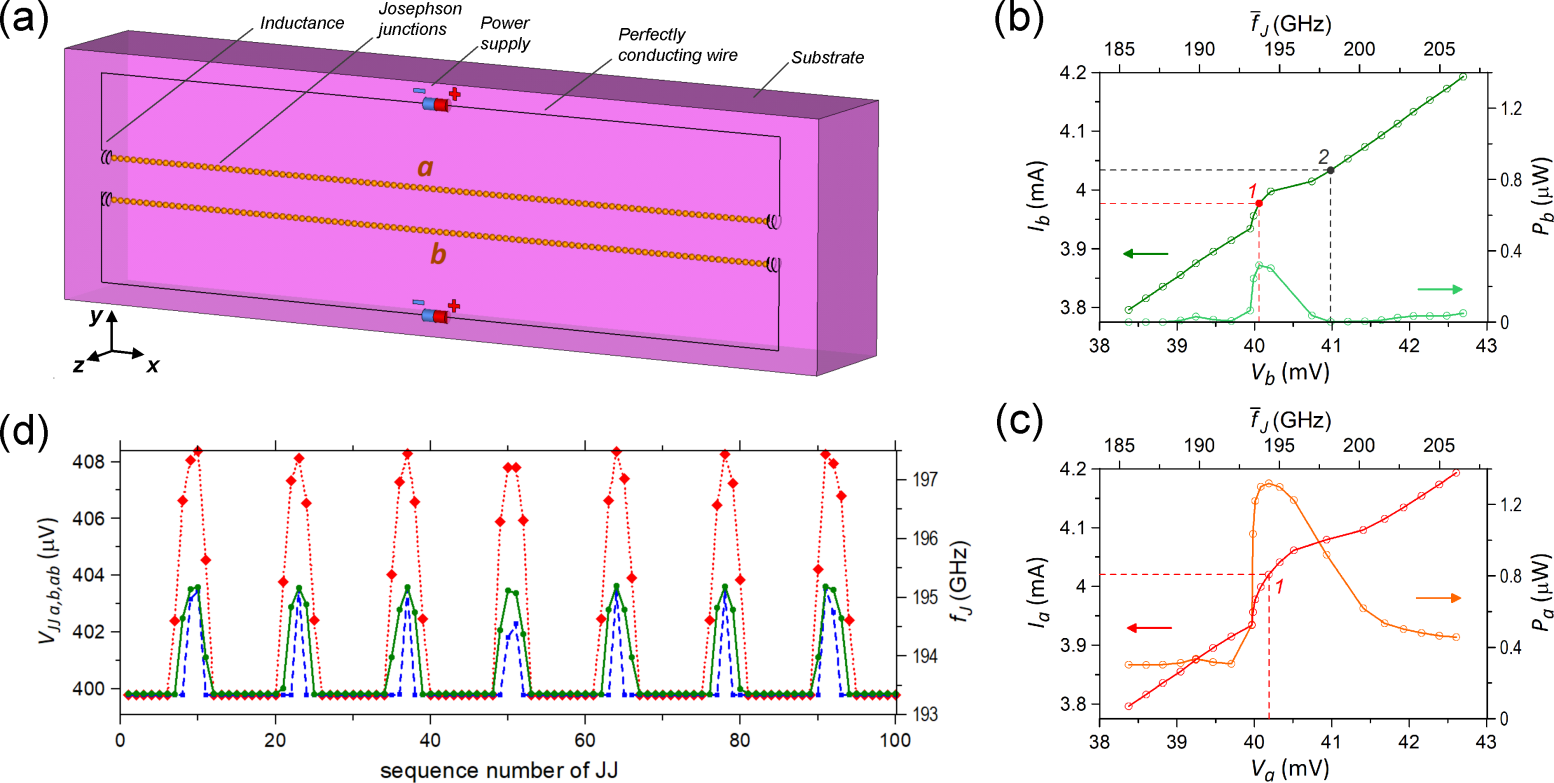
(a) The simulated scheme consisting of two identical JJ arrays on a common substrate. Each array has the form of a line containing 100 JJs. The dimensions of the substrate (*x* × *y* × *z*) are 2.0 × 0.6 × 0.3 mm, and its dielectric permittivity is ε = 12. The inductances have the value 100 pH while the internal resistance of the power supplies is 90 Ω. The junctions are described in the RSJ model [20] with parameters *I*_c_ = 2.5 mA, *R*_n_ = 0.1 Ω, and β = 2. (b) The IVC (left axis) and radiation power (right axis) of array-b when array-a is unbiased. The radiation power reaches the maximum at point 1, which corresponds to the electromotive force 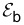 = 398 mV. Point 2 is taken for the analysis in Figure 7a,b. (c) The IVC (left axis) and radiation power (right axis) of array-a when array-b is biased at 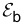. At the indicated bias point 1 the radiation power reaches the maximum. (d) Distribution of the dc voltage in the JJs along array-b when array-a is unbiased (green solid line with circles) and along array-b (blue dashed line with squares) and array-a (red dot line with diamonds) when array-a is biased at the bias point 1 indicated in panel (c). In all cases, array-b is biased at point 1 in panel (b).

The experimental results presented above show that phase locking of two large JJ arrays is a complex phenomenon, which cannot be reduced to a simple constructive interference of two independent sources. For a better understanding of the phase locking dynamics, we perform numerical modelling. Figure 6a demonstrates the general view of the considered model for on-chip synchronization. It contains two identical JJ arrays arranged on a common substrate with a dielectric permittivity of ε = 12, close to that of silicon. The lateral dimensions of the substrate are 2 × 0.6 mm while the thickness is 0.3 mm. We chose such a narrow substrate to avoid excitation of transverse resonant modes inside the substrate. The substrate is surrounded by vacuum, which is terminated by a perfectly matched layer (PML) to cancel back reflection. The PML conditions, in fact, simulate the walls in an anechoic chamber [19].

The circuit of each JJ array has the form of a rectangle with *L* = 1.8 mm in length and 0.2 mm in width (Figure 6a). The long side close to the center of substrate contains *N* = 100 JJs and two identical inductances at the ends. They are needed to electromagnetically decouple the lines with junctions from other parts of the circuits. A power supply of each circuit is located in the middle of the opposite long side. All lumped elements are connected by ideal conductors located at the edges of mesh. The gap between two arrays is 0.1 mm.

The JJs are described by the RSJ model [20]. The corresponding equations of junction dynamics are solved self-consistently with Maxwell equations, which are calculated by the finite-difference time-domain (FDTD) method [21], as described in [8,12,22]. We used the following junction parameters: *I*_c_ = 2.5 mA, normal resistance *R*_n_ = 0.1 Ω, and McCumber parameter β = 2. These parameters are consistent with experimental data for Nb/NbSi/Nb junctions (Figure 2, Figure 5b,c). The inductances are equal to 100 pH while the internal resistance of the power supplies has the value of 90 Ω. The latter allows for measurements of IVCs close to the regime of constant bias current. However, the electromotive force of the power supply 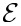 is, in fact, the primary source of biasing. The algorithm of the numerical calculations allows one to obtain both transport and radiation characteristics of the lines with JJs. A more detailed description of the model and the calculation procedure can be found in [8,22].

Figure 6b shows a part of the IVC of array-b and the corresponding dependence of the radiation power *P*_b_(*V*_b_) for an inactive array-a. The power *P* is calculated by integration of the radiation pattern at the specific frequencies where the maximum in the spectrum of the ac current averaged over the JJs is observed [22]. This and subsequent simulations are performed for an upward bias sweep in a range of *V* that is 1.5–1.7 larger than the characteristic voltage *V*_c_ = *I*_c_*R*_n_*N* = 25 mV. As seen from Figure 6b, in this range the form of the IVC is close to a straight line excluding the range *V* ≈ 40–41 mV, where a current step is observed. The step amplitude is Δ*I* = 0.08 mA, and the lowest differential resistance is *R*_d_ = 2.8 Ω. The radiation power increases abruptly at the step and reaches the maximal value *P*_b_ = 0.32 μW at *V*_b_ = 40.06 mV, corresponding to an averaged Josephson frequency of 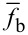 = 193.7 GHz. The indicated value *V*_b_ corresponds to the electromotive force of the power supply 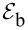 = 398 mV. Array-b was then constantly biased at 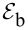 for the subsequent analysis of inter-array coupling.

The results of the simulation with two biased arrays are represented in Figure 6c. They show the calculated IVC of array-a at constantly biased array-b as well as the calculated radiation power. It can be seen that, similar to the experimental observations (Figure 3a,c, Figure 4a, and Figure 5c), the step in the IVC becomes more pronounced compared to the previous simulations in Figure 6b. The amplitude has doubled, Δ*I* = 0.17 mA, and the differential resistance decreased by nearly 5.5 times. The total emitted power *P*_ab_(*V*_a_, *V*_b_) has a nonzero offset *P*_ab_(*V*_a_ = 0, *V*_b_) ≈ 0.32 μW, corresponding to the power of the individually biased array-b. The maximum total power of *P*_ab_ = 1.32 μW is observed at *V*_a_ = 40.19 mV (point 1 in Figure 6c). The gain factor is *g* ≃ 2.1. This value is consistent with the experimental values reported in Table 1 and Table 2.

Figure 6d shows the distribution of the average dc voltage on the JJs for both simulations. The green line is are for the individually biased array-b and the red/blue line is for the collectively biased array-a and array-b. Here, we can clearly see a signature of standing waves along the arrays. We can see eight flat regions with almost equal junction voltages and frequencies 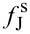 = 193.3 GHz. These junctions are in the antinodes of the cavity mode and are synchronized by the EM field of the standing wave oscillating at the frequency 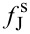.

The junctions located at the nodes of the resonant mode are asynchronous. This means that their Josephson frequencies 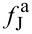 differ from the radiation frequency 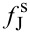, actually 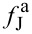 *>* 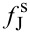. A similar pattern was obtained in [8], but that inequality was opposite, that is, 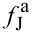 *<* 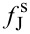. As follows from [8], the latter relation occurs at β ≪ 1. The small value of β also establishes the inverted shape of current steps in the IVC compared to that shown in Figure 6b,c. It can be shown in the same manner as in [8] that the relation between *f*_J_ of synchronous and asynchronous junctions as well as the shape of current steps changes to the opposite at β *>* 1. Also note that the difference of asynchronous regions for array-a and array-b, which is clearly seen in Figure 6d, is caused by the different biasing sequences and the corresponding history-dependent dynamics.

## Discussion

Our experimental data and numerical simulations demonstrate that large JJ arrays can be effectively coupled to each other, resulting in a coherent superradiant enhancement of the emission power. The amplification is observed in the frequency range of 100–200 GHz, both for arrays on a common substrate and for arrays on different substrates formed in a stack. We explain this effect by the interaction between JJ arrays via an EM field. This field is excited along the surface of the substrate as well as inside the substrate. The amplification tends to grow with an increase of the frequency although the overall radiation power decreases. Similar results were obtained for Bi_2_Sr_2_CaCu_2_O_8+_*_x_* mesa structures in [14–15]. In [14], three simultaneously biased mesas emit a high power of 610 μW while each mesa alone emits a maximum of 120 μW. Following our terminology, this corresponds to a gain factor of *g* = 1.7. In [15], the interaction between two mesas has been revealed via the study of polarization of EM emission. Similar to the present work, the obtained data allows one to conclude that such intrinsic JJ arrays have a mutual coupling through the common substrate.

As described in the Introduction, the simple constructive interference of two oscillators, be it single JJs [23] or arrays, should result in a gain of *g* 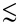 2. However, in this work, we observe also significantly larger gains. Although this clearly indicates that coherent emission from both arrays takes place, it also indicates that additional more complex phenomena are involved. The clue to understanding is provided by the inset in Figure 3a, which demonstrates that phase locking of the two arrays leads to enhancement of the oscillation amplitude in array-a. From Figure 2, we observe that resonant steps in individually biased arrays are more pronounced in the inner array-b than in the outer array-a. At higher bias, steps in array-a are almost invisible. Let us suppose that, initially, only array-b is synchronized at the cavity mode and emits radiation while array-a is not synchronized and, therefore, practically not emitting. In this case, if inter-array coupling totally synchronizes array-a, then the gain factor would become four. This explanation is consistent with the observation that *g >* 2 is observed for higher steps (Table 1 and Table 2), which are less pronounced in the individually biased array-a. This is also confirmed by numerical simulations where we also observed *g >* 2. Therefore, the gain is caused both by the coherent superradiant effect and by the enhancement of the oscillating EM field in each array. The latter is an additional factor that explains why/how the gain factor could be larger than two. The key is that, in our case, the interaction takes place between large arrays with many oscillating junctions. This is a much more complex phenomenon than locking of two oscillators. Here, a mutual synchronization of the two arrays assists also in better internal synchronization within each of the arrays.

The simulated voltage profile in Figure 6d clearly indicates that the cavity mode is playing a decisive role for synchronization of the array. Junctions in the antinodal regions are phase-locked by the driving EM field of the cavity mode. In the nodal regions, the driving force is very small, and, therefore, JJs are unsynchronized there. These asynchronous nodal regions make a vanishingly small contribution to the radiation power (see Appendix). Therefore, the overall emission spectrum remains very sharp and is practically not influenced by the voltage/frequency deviation at nodal JJs. In fact, it is the cavity mode in the electrode, rather than individual junctions, that is causing the emission. The role of the JJs is just to excite and pump energy into the mode.

We have observed similar coherent gains for the on-chip case with small, 4 μm (sample-1), and significantly larger, 238 μm (sample-2), separation between the arrays as well as for off-chip measurements on different chips with even larger separation ∼400 μm. This demonstrates that arrays can effectively interact at a fairly long range. It would be interesting to study in more detail how the amplification depends on the separation between arrays. We have already access to a suitable Nb array consisting of several subarrays with different distances between each. Hence, the corresponding measurements may be carried out in close future.

In the simulations, we see the same effect of amplification for the total power radiated by JJ arrays at frequencies near 190 GHz. The amplification is comparable to that from the measurements. We present in the Appendix the distribution of power generated by each junction and of the phase shift between ac voltage and ac current. This analysis gives a visual pattern of the considered effect of amplification as well as of the synchronization of JJs.

## Conclusion

We explored experimentally and numerically the EM interaction between large JJ arrays containing more than 300 JJs and having superwavelength dimensions. The studied Nb/NbSi/Nb JJ arrays exhibit strong cavity mode resonances, caused by the formation of standing waves along the whole length of the array [13]. We observed mutual coupling between the arrays both on the same chip and on different chips and at different separations between the arrays. We reported significant coherent amplification of radiation emission when both arrays are brought to the same cavity mode resonance. A coherent gain factor, that is, the ratio of the joint emission power from the two arrays to the sum of powers from individually biased arrays, as large as 2.9 was observed. This is well beyond the limit of two, characteristic for the bare constructive interference of two oscillators. The large gain factor indicates that additional effects are taking place. The key is that, in our case, the interaction takes place between large arrays with many oscillating junctions. This is a much more complex phenomenon than locking of two oscillators. Here, a mutual synchronization of the two arrays assists also in better internal synchronization within each of the arrays. This facilitates gains larger than two. This conclusion has been supported by the performed numerical simulations.

Finally, we note that the effect of coherent amplification of radiation from several coupled JJ arrays depends on a number of geometrical factors and material parameters. Those should be accounted for in the design and fabrication of large JJ arrays. Proper design, in which this effect is maximally manifested, allows for increasing the output radiation power, which will facilitate the implementation of JJ arrays in practical applications.

## Appendix: Additional Information about Numerical Simulations

The numerical algorithm allows for calculating also the energy parameters for all JJs that can facilitate the study of synchronization of junctions in the arrays (Figure 6a). We can define the work of the *n*-th JJ under an EM field per unit time, or the generated power, as *P_n_* = −Re(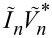)/2 = 
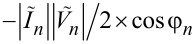
, where φ*_n_* is the phase shift between 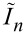 and 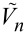, that is, φ*_n_* = arg(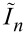) − arg(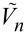). As well as for the radiation power *P*, these amplitudes are taken at the frequency corresponding to the maximum of 
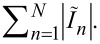


**Figure 7:**
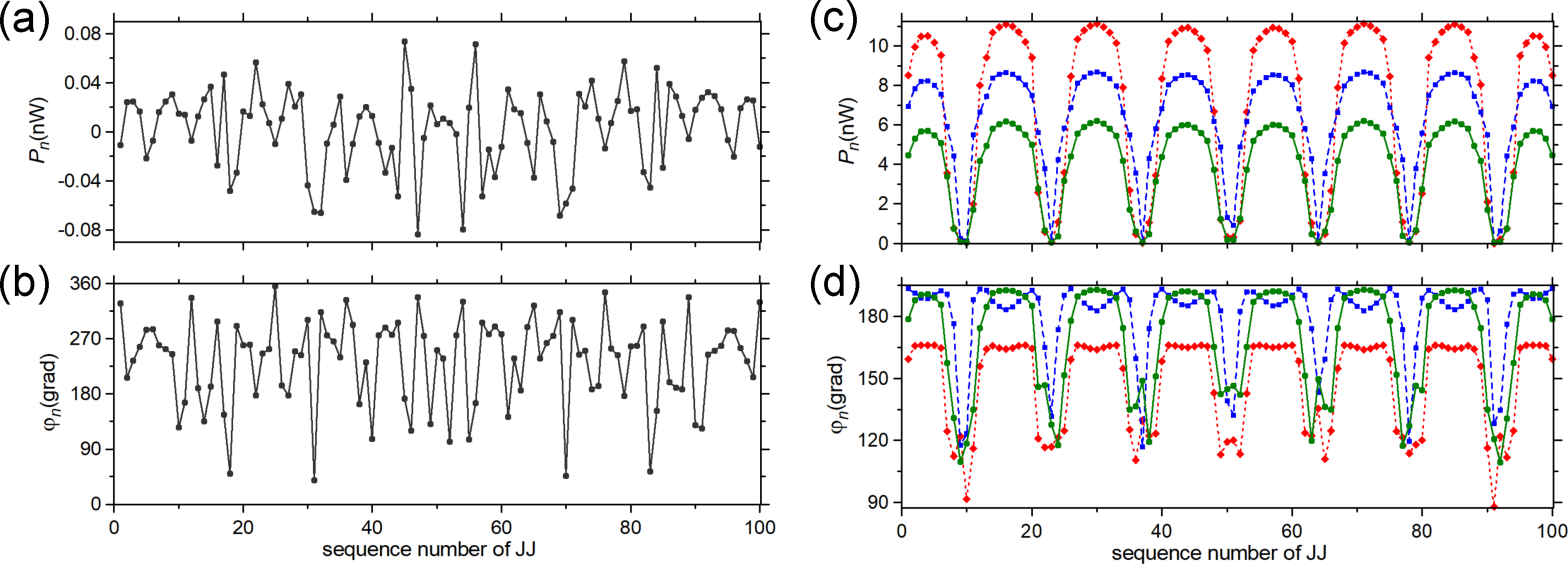
(a, b) Distribution of work of JJs under the EM field (a) and of the phase shift between ac voltage and ac current in the junctions (b) along array-b when array-a is unbiased, and when array-b is out of the current step (point 2 in Figure 6b). (c, d) Distribution of work of JJs under the EM field (c) and of the phase shift between ac voltage and ac current in the junctions (d) along array-b when array-a is unbiased (green solid line with circles) and along array-b (blue dashed line with squares) and array-a (red dot line with diamonds) when array-a is biased. The bias points for the arrays are within the current steps of the IVCs (points 1 in Figure 6b,c).

As seen from Figure 7a, if array-a is inactive and array-b is biased to the point 2 in Figure 6b, out of the current step, then *P_n_* is distributed along array-b rather chaotically taking values of both signs. Almost half of the junctions have a negative sign of *P_n_*. This means that the field does a positive work under these junctions. Hence, these junctions operate as consumers, not as generators. The total generated power in the array is only *P* = 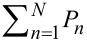 = 0.3 nW. As seen from Figure 6b the radiation power in point 2 is also practically zero on the scale of microwatts. The phase shift φ*_n_* also has a chaotic character taking values in a wide range (Figure 7b). For 77 junctions, φ*_n_* ranges from 180° to 360°. Thus, the differential impedance *Z* of most of the junctions acquires a capacitive character.

When array-b is biased within the current step, and array-a is inactive (point 1 in Figure 6b), the distributions *P_n_* and φ*_n_* along array-b become structured. As seen from Figure 7c,d, they have an oscillation form similar to the distribution of the dc voltage in JJs (Figure 6d). Therefore, *P_n_* and φ*_n_* exhibit, here, the resonant mode excited in the array. As seen from Figure 7c, the junctions in the nodes do a significantly smaller work under the field compared to the junctions in the antinodes, which generate a power of *P_n_* = 4–6 nW. Regarding the phase shift, the junctions in the antinodes have φ*_n_* 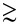 180° (Figure 7d), which is characteristic for a generator with small capacitance. However, when moving towards the nodes, φ*_n_* decreases down to 110–120°, that is, the differential impedance *Z* of the JJs acquires an inductive character.

Similar patterns of *P_n_* and φ*_n_* are observed for both arrays when array-b is biased within the current step (point 1 in Figure 6c). However, the junctions in the antinodes of the resonant mode now generate a larger power: *P_n_* = 7–9 nW for array-b and *P_n_* = 9–11 nW for array-a (Figure 7c). Moreover, the range of junctions that do a large work under the field slightly widens in each antinode. This is in agreement with the conclusion made from the radiative analysis about the amplification of Josephson radiation when two arrays are biased (Figure 6b,c). Each array does a larger work under the field when two arrays are simultaneously biased compared to the case in which only one array is active. Calculating the total generated power *P* for all three cases in Figure 7c, we obtain 0.4 μW from array-b with inactive array-a and 1.35 μW from the arrays when they are both biased. These values are slightly less than those of the radiation power *P*_b_ and *P*_ab_ (Figure 6b,c) calculated by the integration of radiation pattern. This decrease can be caused by dissipations of the radiation in the active loads of the power supplies (Figure 6a).

The phase shift φ*_n_* in the arrays undergoes slight changes when two arrays are biased instead of one (Figure 7d). In each antinode of the mode of array-b, φ*_n_* has two local maxima instead of one with the widening of range where φ*_n_* 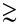 180°. For array-a, the phase shift in the antinodes decreases to φ*_n_* ≈ 160–170°, that is, *Z* of the effectively generating JJs acquires an inductive character.

Keep in mind that *I_n_* is determined by the resonant mode. Thus, it has an equal oscillation frequency throughout each array [8]. Moreover, when the active arrays are coupled, the common resonant mode is formed. Therefore, the data presented in Figure 6d and Figure 7c,d allow one to conclude that the JJs in antinodes of the resonant mode become phase-locked via the common EM field. Such phase locking is fully constructive, that is, all junctions in the antinodes do a positive work under the field. This is what essentially provides the amplification of the Josephson radiation. The present conclusion is in accordance with the data of direct visualisation of the modes presented in [13], in which the global synchronization of the whole JJ array was indicated.
